# Performance and safety evaluation of a cold ablation robot-guided laser osteotome (CARLO) in 28 midface osteotomies

**DOI:** 10.1038/s41598-024-68557-7

**Published:** 2024-11-07

**Authors:** Robert Köhnke, Shih-Jan Chin, Alexandre T. Assaf, Katja Helmbold, Andreas A. Müller, Philipp Juergens, Tobias Wilken, Sibylle Hirsch, Marta M. Morawska, Jan Wolff, Ralf Smeets, Lan Kluwe, Daniel Holzinger, Kurt Schicho, Gabriele Millesi

**Affiliations:** 1 Department of Oral and Maxillofacial Surgery, University Medical Center Hamburg-Eppendorf, University of Hamburg, Hamburg, Germany; 2 Department of Cranio-Maxillofacial Surgery, University Hospital, Basel, Switzerland; 3 MKG Arabellapark - Private Clinic for Oral & Maxillofacial Surgery, Munich, Germany; 4 Advanced Osteotomy Tools AG, Basel, Switzerland; 5 Department of Dentistry and Oral Health - Section for Maxillofacial Surgery and Oral Pathology, Aarhus University, 8000 Aarhus C, Denmark; 6 Department of Oral and Maxillofacial Surgery, Medical University of Vienna, Vienna, Austria

**Keywords:** Orthognathic surgery, 3D-planning, Laser guided osteotomy, Medical research, Health care

## Abstract

The CARLO (cold ablation robot-guided laser osteotome) is a compact device with integrated multiple sensory, steering and safety checking elements. A multi-center study was performed to evaluate the CARLO device for the linear part of midface osteotomy in 28 patients. Feasibility, success rate, safety, performance and experience of the surgeons were assessed and evaluated. All 28 procedures were completed with CARLO without falling back to the conventional methods, giving a technical success rate of 100%. For 27 (96%) cases, procedural success was achieved with cutting lines deviation less than 2 mm. For 25 procedures, the CARLO-cutting was conducted smoothly. In the other 3 cases, some minor difficulties related to the reference markers were reported. For 18 procedures, no change for the cutting path was necessary. For the other 10 cases, cut path was adapted. Intraoperative re-planning was possible, easy and quick without significant delay of the procedures. No CARLO-related adverse events were recorded. Especially, there was no unexpected and unusual bleeding during the CARLO-conducted osteotomy. The time needed for the registration ranged from 1 to 12 min (median = 4). The CARLO-cutting lasted for 5 to 21 min (median = 7). The present study demonstrated feasibility, simplicity, safety, reliability and accuracy of CARLO for the linear part of midface osteotomy.

## Introduction

In cranio-maxillo-facial surgery, digital planning based on the computer tomography and magnetic resonance image data is being increasingly conducted. However, the intervention itself is still performed manually in most cases. This situation leads to frequent discrepancies between the ideal plan and the real outcome^1,2^. Various strategies have been conceived to translate the digital plan directly and precisely into the operation. Navigated osteotomy using parameters of the digital plan is one such strategy^3^, which is however time- and resource-consuming, and requires an enlarged access. Conventional mechanical instruments for cutting hard bone are often not compatible with the precise and fine steering of navigation^4^. Piezoelectric tools provide some improved accuracy and demands less cutting width. Also according to our own experience, duration of piezoelectric cutting is considerably shorter than conventional mechanical ones^5^.

Laser provides another option for navigated osteotomies and solves some weaknesses of piezoelectric cutting. In reference^6^ used a square pulsed Er:YAG laser for minor bone cutting in oral and maxillofacial surgery and have demonstrated a remarkable cutting efficiency without any thermal side effects and damage of adjacent soft tissue structures. However, the lack of depth control and the necessity for careful handling are technical limitations. Several groups have demonstrated similar good performance and tissue healing in osteotomies achieved with a pulsated Er:YAG laser in animal and clinical studies^7–9^.

Due to the lack of depth control and accurate guide, laser-based bone-cutting has been limited to minor surgeries. Especially in the highly complex maxillofacial area, accurate position control is essential. The mechanical force in cutting major bone is large and often causes position shift and direction change of the surgery area. An optical tracking is therefore essential to enable recalculation of parameters in the navigation during surgery^10,11^. However, accumulation of such aiding devices increases the complicity of the technology and makes it impractical around the surgical table. Integrating multiple sensory and steering elements into a compact device with simple and safe protocol therefore is essential for application of laser-based photoablation in “major” bone surgery.

The CARLO (cold ablation robot-guided laser osteotome) is such a device, which implements preoperative plans with a full digital workflow, without requiring the manufacturing of custom-made cutting guides or splints, especially in upper jaw osteotomies. Pre-clinical studies revealed high accuracy especially in retaining preoperatively planned geometries during surgery and unique possibility of functional non-linear cuts. The cold ablation cutting technique enables better preservation of the structure and vitality of the adjacent bone tissue. When compared with piezoelectric cutting, the cold ablation generates less heat. The increased level of automation in navigation further reduces surgeon-dependent workability in accuracy.

In a recent feasibility study^12^ on a 19 years patient, the digital plan for a linear LeFort I midface osteotomy was transferred to the CARLO device which then conducted the linear part of the osteotomy autonomously under direct visual control. The maximum difference was 0.8 mm between the planned and performed osteotomy, with a root-mean-square error of 1.0 mm.

Based on the promising results of the feasibility study, the present prospective multi-centre study was designed and conducted to evaluate the CARLO device for the linear part midface osteotomy in 28 patients. Parameters and features including feasibility (without falling back to conventional tools), success rate, safety, performance and experience of the surgeons were assessed and evaluated.

## Results

### Primary outcomes

#### Technical success

**Figure 1:**
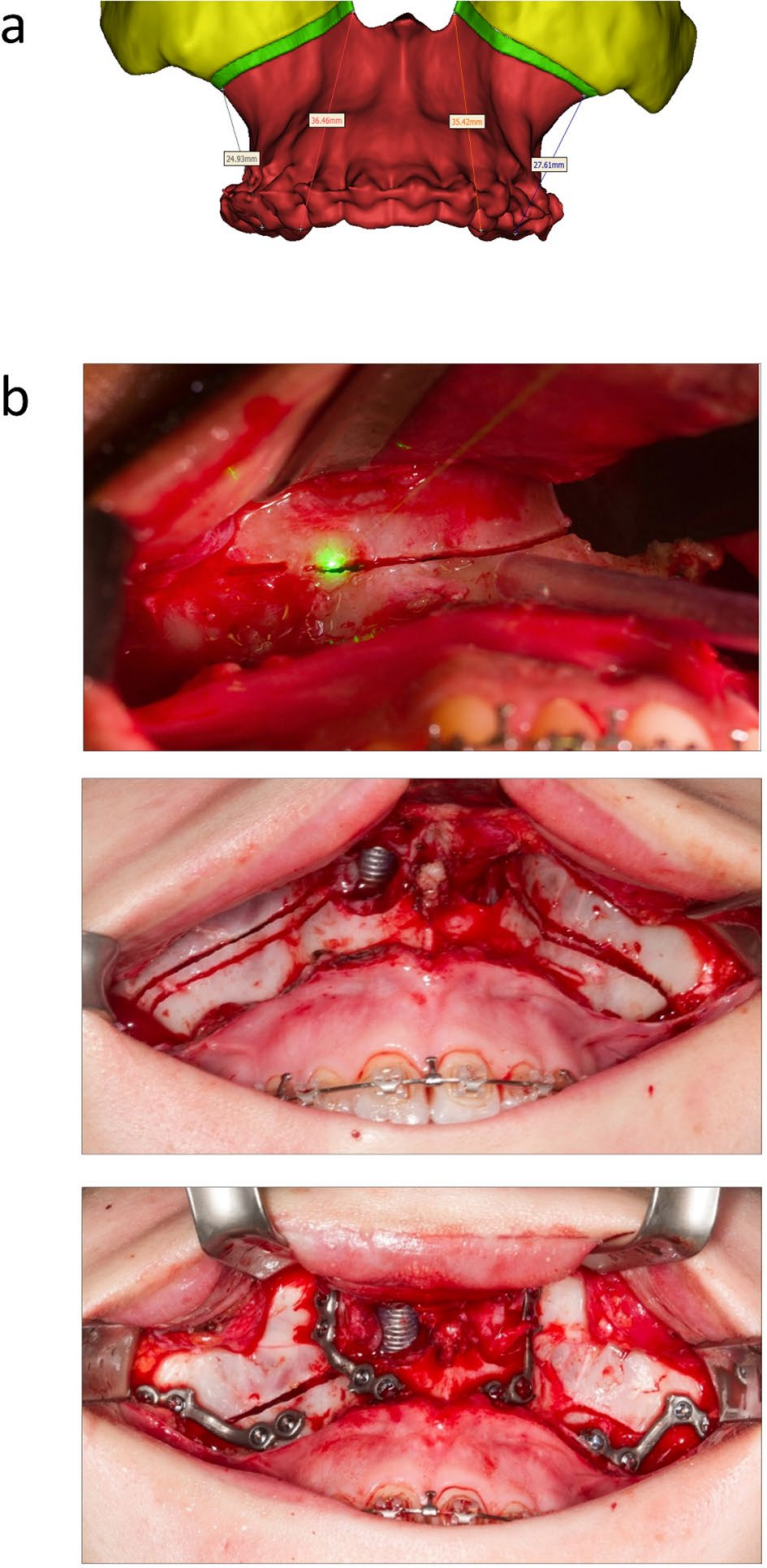
Planning and intraoperative view. (**a**) Typical location of the four preoperative defined locations for the accuracy measurement (tips of the mesio-buccal cuspids of the first molars and canine). Yellow: midface; red: maxilla; green: resected bone, gap. (**b**) Intraoperative view (same patient) of the cutting process, green light: tip of the cutting laser. Middle: Osteotomies that were shown in Fig. 1a, directly after cutting. Below: osteosynthesis of the maxillary correction with patient-specific implants.

For all the 28 procedures, set-up and self-test of the CARLO device were successful (Fig. 1). All osteotomies were completed with CARLO without falling back to the conventional methods (saw, chisel and burr), giving a technical success rate of 100%.

#### Procedure success

**Figure 2:**
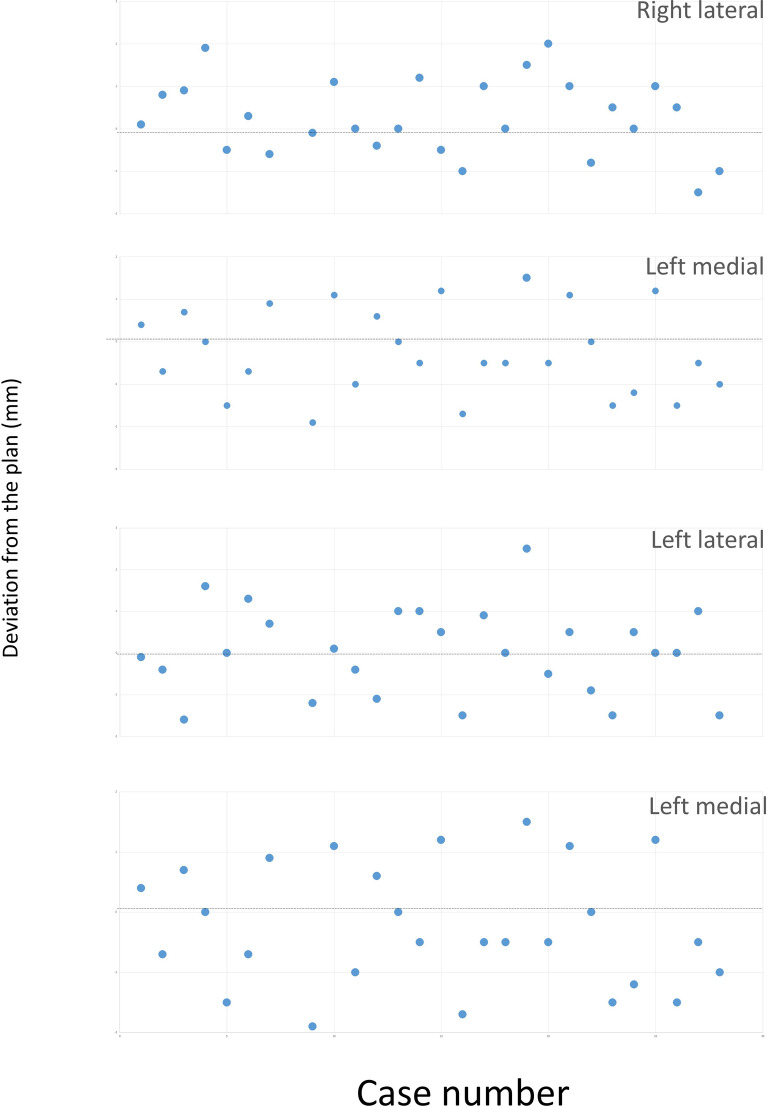
Difference between the planned and the intraoperative measured distance to the reference landmarks.

**Figure 3:**
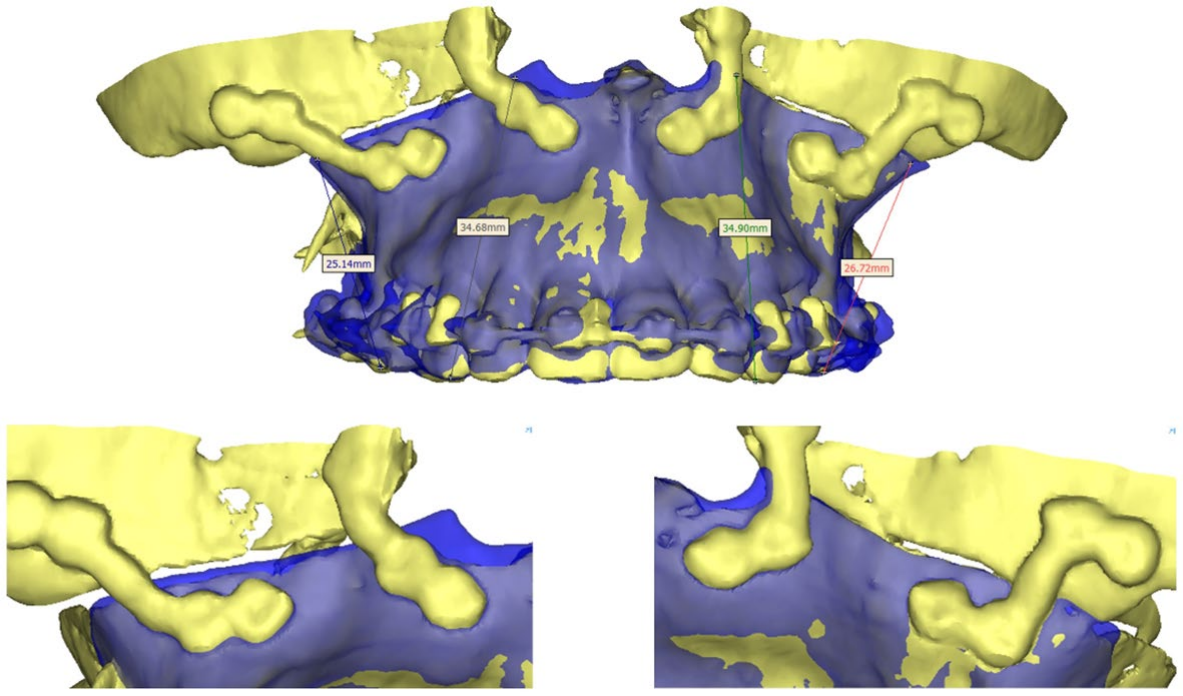
Overlay of preoperative planned, cut maxillary segment (blue overlay) with postoperative outcome of the osteotomy (yellow) for a patient with pre- and postoperative CT images.

For 27 patients out of the 28 valid patients, procedural success was achieved (Figs. 2, 3), corresponding a rate of 96%. The lower end of one-sided 95% exact Pearson-Clopper confidence interval was 84%.

In one case (03-015) out of the 28 valid cases, one (left-lateral) of the 4 cut lines differed 2.5 mm from the corresponding planned line, 0.5 mm exceeding the defined maximum deviation of 2 mm. Therefore, this case was not rated as success. Nevertheless, intraoperative requirements for an interruption were not met and the osteotomy was completed with CARLO.

### Secondary outcomes

#### Difficulties in deploying and using CARLO

For 25 procedures, the CARLO-cutting was conducted smoothly. In the other 3 cases, some minor difficulties were reported, all were related to the reference markers. In one case, the patient reference marker needed to be adjusted (03-001). In the other two cases (04-005, 04-006), there was a collision between the patient marker and the laser-arm. However, these difficulties did not fulfil any of the criteria for interruption and the osteotomy was continued and completed with CARLO.

In one other case, a device-deficiency (03-012) was observed. However, CARLO was controllable for the whole osteotomy procedure and did not lead to any adverse event.

#### Intra-operative adjustment of the cutting path

For 18 procedures, no change nor adjustment for the cutting path was necessary. For the other 10 cases, cut path had to be adapted (Table 1). Intraoperative re-planning of the path was possible, easy and quick without significant delay of the procedures. The re-planned cutting was conducted without any problem.

**Table 1:** Intra-operative changes for the planed cutting lines and reasons.

Patient	Change_right	Reason_right	Chang_left	Reason_left
01-004	3 mm	Decision of the surgeon	No change	n/a
03-012	Adjust cutting line	Anatomical reasons	Adjust cutting line	Anatomical reasons
04-005	Change laser angle relative to surface	Collision with patient marker	No change	No change
04-006	Change laser angle relatives to surface	Laser collides with marker	New registration necessary	Marker movement by laser before pre-visualization. Change angle of laser or laser head not visible by camera new registration necessary
03-001	Change of angle & position	According to intraoperative judgement	No change	n/a
04-003	Change of the cutting angle necessary	Injury to the lip possible	Incision 2 mm after medially extended	Paranasal abutment was not cut by laser
04-002	Lateral extension of the incision is necessary	Cut too short	Extension of the incision line necessary	Cut dorsally too short
03-006	No change	n/a	Extension of cut off line	Extension of cut off line
03-018	No change	n/a	Extension of the cutting line	Anatomical rea-son
04-001	Resetting from 2 mm to caudal	Surgeon`request; aim was 27,5 mm for the distance R 1–3	No change	No change

#### CARLO-related adverse events

**Figure 4:**
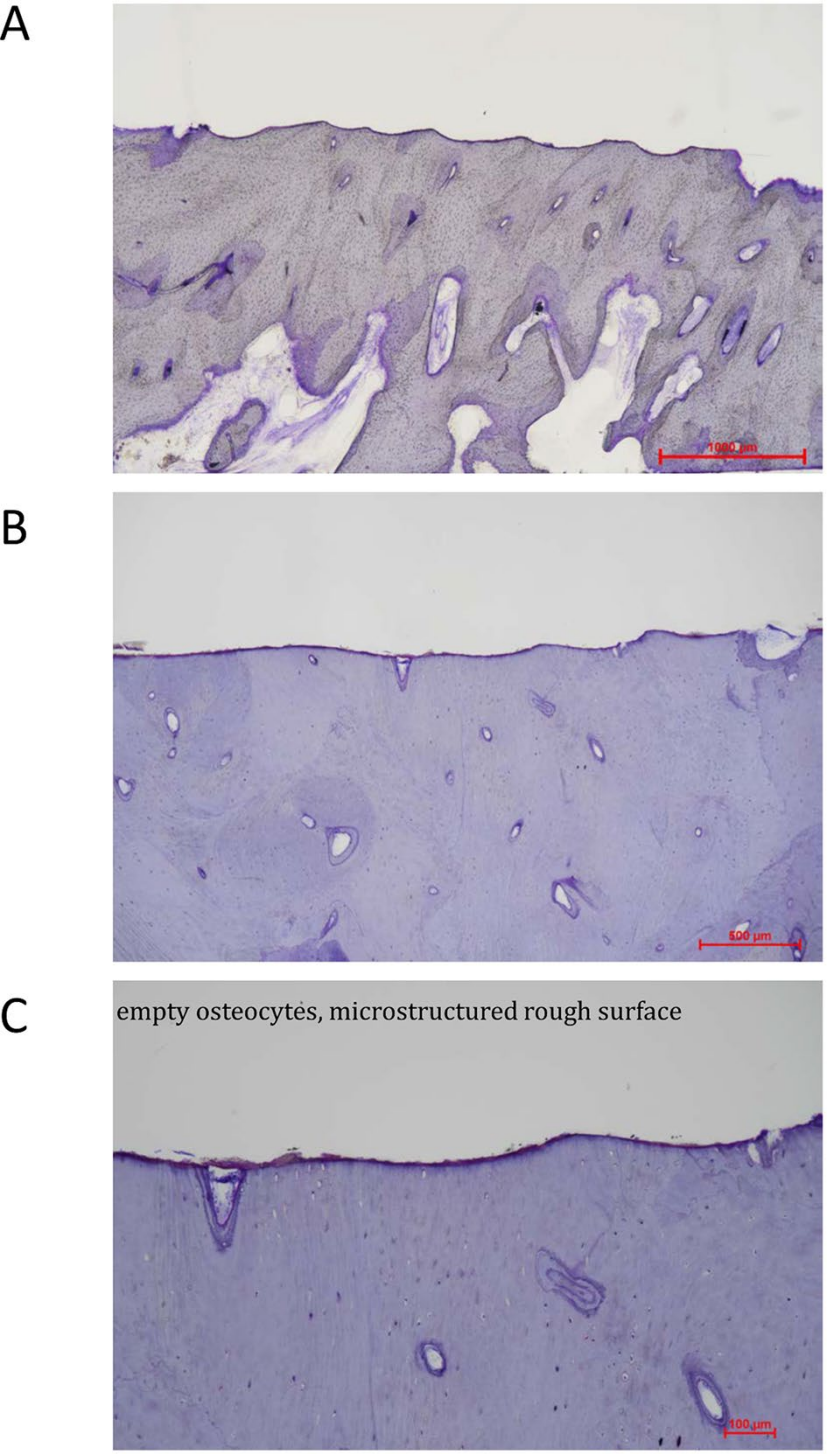
Histological staining of the sinuous line cutting surface.

No CARLO-related adverse events were recorded. Especially, there was no unexpected and unusual bleeding during the CARLO-conducted osteotomy. No damage of the surrounding soft tissue was observed. No heat-related damage was visible on the cutting surface (Fig. 4).

Any general adverse events such as bleeding, swelling, headache and nausea were associated with the anaesthesia and surgery, not directly related to the CARLO. An unexpected auditory and visual hallucination was recorded in a patient with large number of concomitant medications, lacking any link to the CARLO. A blepharospasmus in the right eye was also evaluated as independent of the device and of device-related procedures.

None of the patients showed any significant problem in wound healing, active bleeding, infections or sensitivity loss at time of discharge. Follow-up investigations did not reveal any deterioration in the above mentioned assessment factors.

#### Durations

**Figure 5:**
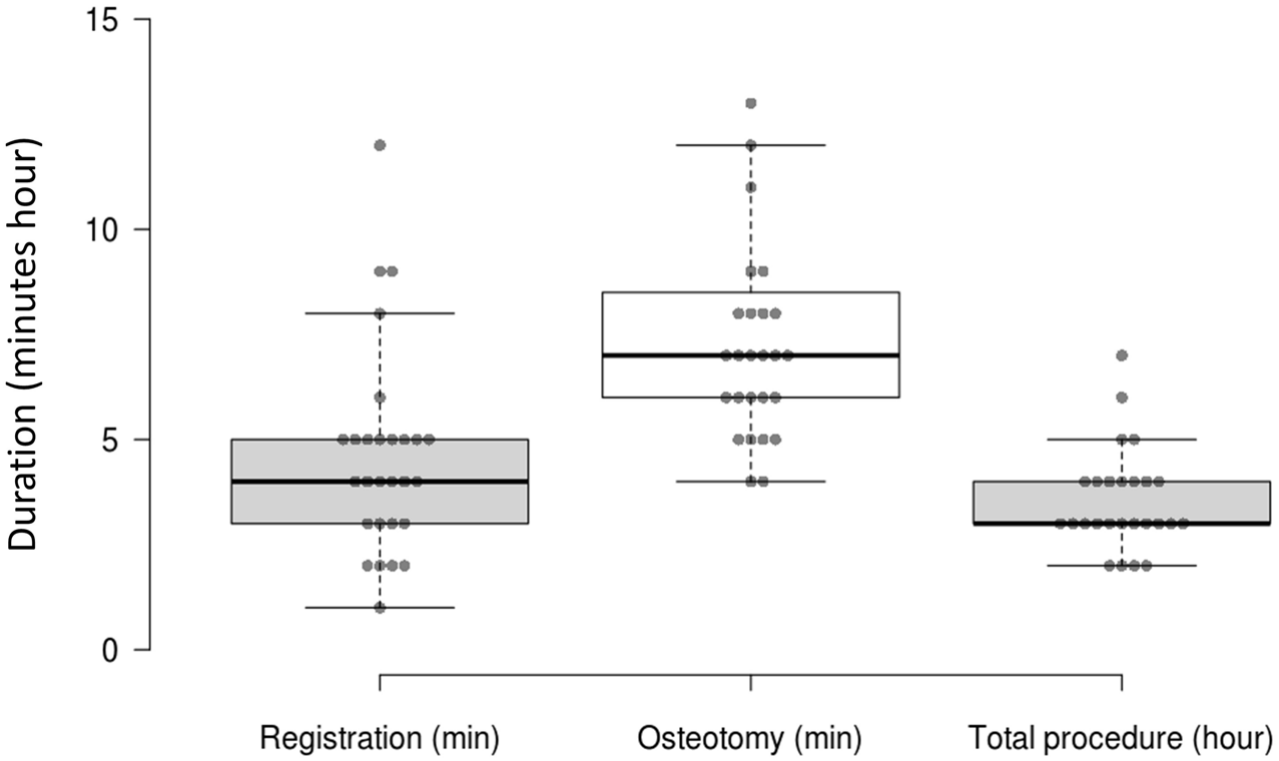
Duration of the CARLO-registration (in minutes), the CARLO-cutting (in minutes) and the whole procedure (in hours). The thick lines in the boxes indicate the medians.

The time needed for the registration ranged from 1 to 12 min (median = 4). The CARLO-cutting lasted for 5 to 21 min (median = 7). However, the time with activated laser was shorter. Considerable time was spent for dealing with the device in a live situation, and for taking photos and videos for documentation purposes. The whole procedure lasted 2 to 7 h (Fig. 5).

#### Further safety outcomes

Neither for patients nor for any of the medical staff CARLO-related injury was recorded.

## Discussion

This multi-centre clinical study demonstrated safe and successful application of the CARLO for the linear part of mid-face osteotomy surgeries. All 28 surgeries could be conducted completely with the CARLO without falling back to standard methods or the necessity for a device interruption. No severe difficulties or significant and unexpected delays were encountered. In 27 out of 28 surgeries and 111 out of 112 measurements, the accuracy was within the defined tolerance of 2 mm, corresponding to a > 99% success rate in accuracy.

In one patient, there was an unexpected minor excess deviation of 0.5 mm at one extremal part of the cutting line. For this patient, only three landmarks were used during registration. Though the recommendation of CARLO is 4, technically it was possible to use 3 landmarks. However, reduced number of landmarks may have reduced the precision of the registration. For the subsequent procedures, the recommendation of 4 landmarks was strictly followed.

An intrinsic weakness of Er:YAG-lasers is the 0.9 mm laser-beam focus which limits the precision. In comparison, the piezoelectric cutting has thickness of 0.1–0.2 mm. Also a conventional cutting aided by 3D-printed surgical guide with osteotomy-line slits can reach high precision. Improving the beam focus is therefore a major remaining issue for Er:YAG-laser for reaching thinner cutting lines. At present, this rather thick cutting line has to be considered in the virtual planning to ensure safe distance to critical tissues.

The study design of an initial pilot-group of four sequential procedures provided an additional safety measure, since all subsequent procedures could be reconsidered or stopped upon any unexpected events or problems. All the 4 procedures were successful and the criteria were met. The main study could therefore be continued with the same protocol.

No CARLO-related injury or any adverse events were recorded. No unexpected contact of the CARLO to the medical staff happed. In contrast to piezoelectric cutting, this cold ablation laser does not generate heat on the cutting surfaces which is expected to favorite the bone healing.

The duration of CARLO cutting is satisfactory and compatible with that of conventional ones. For more than half of the cases, less than 11 min were needed for the CARLO-registration and cutting. Considering the total duration of 2 to 7 h of the whole procedure, the extra 11 min were only marginal. Upon accumulation of experience, this time is expected to be shorter.

**Figure 6:**
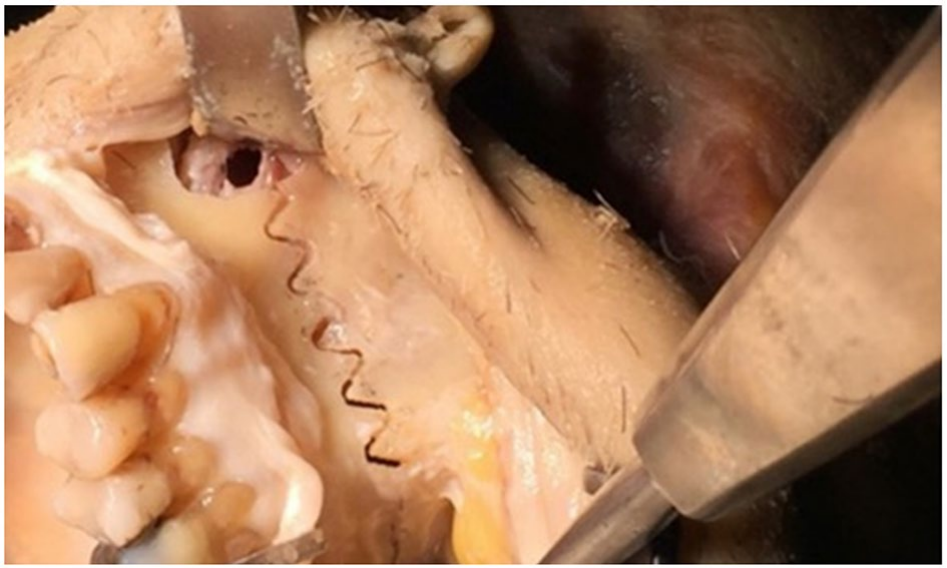
Wave-curved cutting line performed by CARLO in the frontal wall of the maxillary sinus of a cadaver.

Despite the improvement in making the device compact, the device CARLO is still large in size and therefore has its limitation in applications, for example, for small operation rooms. Another drawback of laser-based cutting tools is the high cost that we hope will go down as the technology further develops and the application areas increase. In summary, the present study demonstrated safety and reliability of CARLO in midface osteotomy. With the unique ability to perform osteotomies of any contour, the CARLO will enable non-linear functional cuts (Fig. 6). This is envisioned to simplify the repositioning of the bone, provide a primary stability of the desired bone fixation. The predefined cuts are executed by the laser, and, without manual pressure, additional fracture lines and cracks can be avoided independent of the thickness of bones in the sinus walls for example. Improved bone tissue preservation is envisioned to reduce related complications with grafts and implants. Enhanced tissue vitality also benefit patients suffering from impaired vascularization. Furthermore, functional cuts may spare a second operation to remove osteosynthesis materials. Actually, studies are in progress to assess the feasibility and suitability of the device for other procedures such as BSSO.

## Methods

### Study design

This is a prospective, tri-national, multi-centre, open-label and single arm clinical study, consisted of an initial pilot group of 4 patients and a main study group of 24 patients. Protocol, informed consent and other required documents have been reviewed and approved by Independent Ethics Committees (Ethikkommission der Medizinischen Universität Wien, Ethikkommission Nordwest- und Zentroischweiz (EKNZ), Ethikkommission Ärztekammer Hamburg), and notified by Regulatory Authorities (Bundesamt für Sicherheit im Gesundheitswesen (BASG) Österreichische Agentur für Gesundheit und Ernährungssicherheit (AGES), Swissmedic, Bundesinstitut für Arzneimittel und Medizinprodukte) prior to study initiation. The study was registered under trial registration number NCT03901209, SNCTP000002432 on 03/04/2019. Additionally, the study was registered with the European Database on Medical Devices (CIV-19-02-027204). The study was conducted in accordance with the ICH-GCP Guidelines (Directive CPMP/ICH/135/95) and the Declaration of Helsinki (1964) and subsequent revisions and the ISO 14155.

Patients who met the inclusion criteria and none of the exclusion criteria were defined as eligible for the study. Eligible patients could only be included in the study after providing written informed consent.

The main including criterion is the indication of a midface osteotomy via a transoral approach during orthognathic surgery that can be performed using bilateral straight-cut lines (LeFort I). The detailed inclusion and exclusion criteria are shown in supplement.

In the initial pilot study, 4 interventions were conducted sequentially with a minimum interval of 2 weeks. After each of these surgeries, data including procedure report, intraoperative observation and soft-tissue healing 2 weeks after the surgery were reviewed, and the risk and benefit were evaluated. In case the soft tissue wound healing was uncertain, two more weeks were awaited and the evaluation was repeated. In these 4 weeks cases, bone healing was also confirmed by a 3D imaging. Only upon satisfactory evaluation, the next surgery was planned and carried out.

After completion and satisfactory evaluation of the initial pilot study, the main study was started with the recruitment of 24 patients at the 3 participating centres: General Hospital AKH-Medical University Vienna, University Hospital Basel and University Medical Center Hamburg Eppendorf.

### Imaging for planning and outcome evaluation

For pre-treatment assessment and surgery planning, 3D images were performed and the data were used to plan the osteotomies and the maxilla relocation with the “Mimics” software version 21.0 (Leuwen, Belgium) (Fig. 1a). The output file in the form of a “.stl” was transferred to the CARLO. The safe distance to critical tissues is set as 5 mm, which covers the 0.9 mm thickness of the laser focus.

Postoperatively, 5 patients received a CT while the other 23 patients were controlled using Cone Beam CT (CBCT) following the standard practice at the study sites. CT-data of the 5 cases were used to calculate parameters in 3 directions (X/Y/Z) describing the maxilla position to defined landmarks (e.g. tips of the mesio-buccal cuspids of the first molars, tips of the upper canines, incisor point) that were compared to those in the preoperative plan using the Mimics software (Fig. 1a).

### Procedure

The procedure was as described in detail in the feasibility study for the first patient using CARLO^12^. Briefly, the surrounding soft tissue was protected by Langenbeck Hooks for lip and cheek, and a metal spatula for the nasal entrance (Fig. 1b). A green pre-cutting-light was checked for possible contact with surrounding soft tissue. A reference marker was then attached to the targeted bone and a referencing to the preoperative planning data was performed. Detaching the bone and exposure of the maxillary sinus wall were carried out. An in-situ visual check was carried out on the cutting trajectory generated by a green, low-energy aiming beam. If the surgeon was satisfied with the pre-visualized plan, a confirmation was given. Otherwise, the surgeon could redefine the cutting line in the plan and then confirm in regards of proximity of roots or height of osteotomy site for example. Once the cutting path is confirmed the surgeon initiates and controls the osteotomy with the handheld switch.

After the successful execution of the linear part of the osteotomy, the distances between 4 reference points at the teeth and the cutting lines were measured using a surgical compass. A deviation of less than 2 mm between the distances on the preoperative plan and the intraoperative measurement was defined as success in accuracy.

The CARLO device was removed from the operation table and the subsequent maxillary mobilization, fixation and wound closure were carried out following the standardized protocols of each site.

### Follow up examinations

Swelling, redness, active bleeding, infection at the surgical site and sensitivity disturbance were controlled at discharge, 2, 4 and 6 weeks after the surgery (Table 2). Also between the scheduled follow-ups, consult and examination was available for the patients especially regarding wound survey, osteotomy-related injuries, other adverse events and other related or unrelated concomitant medication.

**Table 2:** Study schedule and acquired parameters.

Study periods	Screening	Pre- treatment	Intervention	Follow-up period10				End-of-the-study (LPLV)
Visit	SC	PT	V1	V2	V3	V4 (Individual EoS)	V5 (In-divid-ual EoS)	
Time (hour, day, week)	Max. 3 months before surgery (-14 days to day -1)	Max. 2 weeks before surgery (-14 days to day -1)	0	Day 7 ± 2 or discharge*, whatever comes first	14 ± 4 days	28 ± 4 days	42 ± 4 days	After approx. 9month11
Information and informed consent	X							
Demographics	X							
Medical history	X							
In-/ exclusion criteria	X	X	X4					
Physical examination	X		X4	X	X	X	X	X
Allocation of subject number	X							
Vital signs	X		X	X	X	X		X
Laboratory tests		X	X	X				
Pregnancy test1	X	X	X4					
Imaging (CT or CBCT)2		X3		X6		(X)7 only for centers AT and CH	X7 only for cen-ter DE	
3D planning of surgery		X						
CMF-surgery			X					
Assessment/ documenta-tion of device related in-juries			X	X	X	X		X
Assessment bleeding, swelling & pain			X	X				
Assessment soft tissue re-covery (wound healing mucosa/ infections)					X	X		X
Removal of stitches					X			
Concomitant therapy, in-tervention	X	X	X	X	X	X		X
Adverse event collection			X*	X*	X*	X*	X*8	X
Completion of case re-port forms	X		X	X	X	X	X	X
Inform about follow-up phase until LPLV8						X		

### Ethics approval and consent to participate

Protocol, informed consent and other required documents have been reviewed and approved by Independent Ethics Committees and notified by Regulatory Authorities prior to study initiation.

## Supplementary Information

Supplementary Information.

## Data Availability

The data that support the findings of this study are available on request from the corresponding author, [RK]. Restrictions apply to the availability of these data, which were used under license/ intellectual property protection for this study.

## Abbreviations

CARLO: Cold ablation robort guided laser osteotome
Er:YAG: Erbium-doped yttrium aluminium garnet laser
STL: Standard tessellation language
CT: Computer tomography
CBCT: Cone beam computer tomography
RMS: Root mean square
