# Supplementary material for: Performance and safety evaluation of a cold ablation robot-guided laser osteotome (CARLO) in 28 midface osteotomies
Source: Sci Rep. 2024 Nov 7;14:27160. doi: 10.1038/s41598-024-68557-7 (PMC11544146; doi:10.1038/s41598-024-68557-7)
Supplement: Supplementary file 1 — Supplementary Information. [file 41598_2024_68557_MOESM1_ESM.docx]

**Performance and Safety Evaluation of a Cold Ablation Robot-Guided Laser Osteotome (CARLO) in 28 Midface Osteotomies**

**Supplementary information**

**Enrolment criteria**

**Inclusion**

1 Indications for performing midface osteotomies using a transoral approach and suitability for use in CARLO®

2 Surgical relocation of the maxilla is indicated, using a midface osteotomy via a transoral approach that can be performed using bilateral straight-cut lines (LeFort I). Patients for whom a concomitant re-positioning of the mandibula or other corrections are indicated may be included in the study, as long as these corrections do not affect the repositioning of the maxilla that is being assessed; these additional osteotomies will not be performed using the CARLO®

3 Patient is willing and able to attend all scheduled visits and comply with all study procedures

4 Aged ≥18

5 Ability to understand and give study specific informed consent

6 Written informed consent obtained from patient

7 Proven completion of the facial growth

**Exclusion**

1 Female patients who are pregnant, breast-feeding or are planning to become pregnant during the study

2 Other patients who are vulnerable, such as adults lacking the capacity in the consent procedure, patients in emergencies

3 Known or suspected non-compliance, drug or uncontrolled alcohol abuse

4 Presence of any condition or abnormality that in the opinion of the investigator would compromise the safety of the patient or quality of the data

5 The patient is participating in, or intends to participate in another investigational drug or device clinical trial within 12months after enrolment

6 Enrolment of the investigator, his/ her family members, employees and other dependent persons

7 Missing indication for orthognathic surgery

8 Patients with bleeding diathesis or coagulopathy

9 Patients with increased perioperative airway risk factors due to anatomical structures, which limits usage of endotracheal intubation

10 Patients with intolerance or hypersensitivity to local anesthetics

11 Patients with consumptive/ malignant primary disease (e.g. renal failure, hepatic dys-function, severe heart failure, etc.) and a life expectancy of < 6months

12 Patients that have an odontogenic osteomyelitis

13 Patients that have a diagnosed bone metabolism disorder e.g. osteoporosis, osteomala-cia, paget’s disease, renal osteodystrophy, parathyroid disorders

14 Patients who have received or are receiving antiresorptive therapy (bisphosphonates)

15 Patient has previously undergone radiotherapy in the region of the intended osteotomy

16 Patients with impaired wound healing, for example due to type II diabetes

At sites Switzerland and Austria:

17 Vulnerable persons such as minors and persons lacking capacity in the consent proce-dure are excluded from this study

At site Germany:

18 Patients with a life-expectancy of <12 months

19 Patients with an anamnesis of any fracture within the location of the surgical intervention

**Drop Outs**

Early drop outs that occurred for reasons independent to the CARLO® intervention were re-placed. In order to maintain the power of this study, possible drop-outs were replaced by ex-tending the recruitment as needed. This might only be the case if during surgery the operation could not be finished by using the CARLO® or a piezo device as well (e.g. the patients had a heart attack). Also dropped out patients were followed up in a regular way until end of study.

**Table S1 Study Schedule and acquired parameters**

| **Study Periods** | **Screening** | **Pre-**  **Treatment9** | **Intervention** | **Follow-Up Period10** |  |  |  | **End-of-the-Study (LPLV)** |
| --- | --- | --- | --- | --- | --- | --- | --- | --- |
| Visit | SC | PT | V1 | V2 | V3 | V4 (individual EoS) | V5 (in-divid-ual EoS) |  |
| Time (hour, day, week) | Max. 3months before surgery (-14days to day -1) | Max. 2weeks before surgery (-14days to day -1) | 0 | Day 7 ± 2 or discharge*, whatever comes first | 14 ± 4days | 28 ± 4days | 42 ± 4 days | After approx. 9month11 |
| information and informed consent | X |  |  |  |  |  |  |  |
| Demographics | X |  |  |  |  |  |  |  |
| Medical history | X |  |  |  |  |  |  |  |
| In-/ exclusion criteria | X | X | X4 |  |  |  |  |  |
| Physical examination | X |  | X4 | X | X | X | X | X |
| Allocation of subject number | X |  |  |  |  |  |  |  |
| Vital signs | X |  | X | X | X | X |  | X |
| Laboratory tests |  | X | X | X |  |  |  |  |
| Pregnancy test1 | X | X | X4 |  |  |  |  |  |
| Imaging (CT or CBCT)2 |  | X3 |  | X6 |  | (X)7 only for centers AT and CH | X7 Only for cen-ter DE |  |
| 3D planning of surgery |  | X |  |  |  |  |  |  |
| CMF-surgery |  |  | X |  |  |  |  |  |
| Assessment/ documenta-tion of device related In-juries |  |  | X | X | X | X |  | X |
| Assessment bleeding, swelling & pain |  |  | X | X |  |  |  |  |
| Assessment soft tissue re-covery (wound healing mucosa/ infections) |  |  |  |  | X | X |  | X |
| Removal of stitches |  |  |  |  | X |  |  |  |
| Concomitant therapy, in-tervention | X | X | X | X | X | X |  | X |
| Adverse event collection |  |  | X* | X* | X* | X* | X*8 | X |
| Completion of case re-port forms | X |  | X | X | X | X | X | X |
| Inform about follow-up phase until LPLV8 |  |  |  |  |  | X |  |  |

*(X) Completed when needed – details provided in text. X* Zero or more copies of the form, if relevant event or information is to be captured. 1For Women of childbearing age only: serum βhCG or urine dip stick. 2For pre-treatment assessment and surgery planning, a CT-scan is performed; postoperatively, either a Conical Beam CT (CBCT) is normally sufficient. Recommendation for planning CT are provided; other imaging procedures follow standard practice at the study site. 3Recommended CT parameters for procedure planning are pro-vided in the CIP. 4These procedures may be carried out 1day before the surgery (in patients already hospitalized for the surgery) or on the day of the surgery. 5If the patient is only intro-duced 2weeks before the planned surgery date, the screening visit and pre-treatment visit can be conducted combined. 6Control CBCT Scan for review at V2 can be done any time after surgery and day 7/ discharge. 7If there were signs of a delayed wound healing at visit 3 and persisted, an X-ray exam (Conical Beam CT) will be performed. In the absence of a complete mucosal healing, this examination serves to identify whether there are any signs that bone healing is non-physiological. 8After EoS visit of each individual patient, safety moni-toring will go on for the duration of the study until LPLV. Clinical observations relevant to the osteotomy that may be made during ongoing treatment follow-up, or an unscheduled visit initiated by the patient, will be recorded in the CRF, if applicable.*

*9 Pretreatment visits may be combined with screening or intervention visit, depending on the timing*

*10 In addition, there might be unplanned visits for patients coming to the hospital between scheduled visits. 11This duration can be extended, for instance if recruitment is suspended to evaluate trial interruption criteria (see also 8.6.1Suspension of the clinical study* for trial suspension criteria)
